# Processed Electroencephalogram-Based Monitoring to Guide Sedation in Critically Ill Adult Patients: Recommendations from an International Expert Panel-Based Consensus

**DOI:** 10.1007/s12028-022-01565-5

**Published:** 2022-07-27

**Authors:** Frank A. Rasulo, Philip Hopkins, Francisco A. Lobo, Pierre Pandin, Basil Matta, Carla Carozzi, Stefano Romagnoli, Anthony Absalom, Rafael Badenes, Thomas Bleck, Anselmo Caricato, Jan Claassen, André Denault, Cristina Honorato, Saba Motta, Geert Meyfroidt, Finn Michael Radtke, Zaccaria Ricci, Chiara Robba, Fabio S. Taccone, Paul Vespa, Ida Nardiello, Massimo Lamperti

**Affiliations:** 1 Department of Anesthesiology and Intensive Care, Spedali Civili Hospital, Brescia, Italy; 2 Department of Surgical Specialties, Radiological Sciences, and Public Health, University of Brescia, Brescia, Italy; 3 Institute of Biomedical and Clinical Sciences, University of Leeds, Leeds, UK; 4 Institute of Anesthesiology, Cleveland Clinic, Abu Dhabi, United Arab Emirates; 5 Department of Anesthesia and Intensive Care, Erasme Hospital, Universitè Libre de Bruxelles, Brussels, Belgium; 6 Department of Anaesthesia and Intensive Care, Cambridge University Hospitals NHS Foundation Trust, Cambridge, UK; 7 Department of Anesthesia and Intensive Care, Istituto Neurologico C. Besta, Milan, Italy; 8 Department of Anesthesia and Intensive Care, Careggi University Hospital, Florence, Italy; 9 Department of Anesthesiology, University Medical Center Groningen, Groningen, Netherlands; 10 Department of Anesthesia and Intensive Care, University of Valencia, Valencia, Spain; 11 Division of Stroke and Neurocritical Care, Department of Neurology, Northwestern University, Evanston, IL USA; 12 Department of Anesthesia and Intensive Care, Gemelli University Hospital, Università Cattolica del Sacro Cuore, Rome, Italy; 13 Department of Neurocritical Care, Columbia University Irving Medical Center, New York, NY USA; 14 Critical Care Division, Montreal Heart Institute, Université de Montréal, Montreal, Canada; 15 Department of Anesthesiology and Critical Care, Universidad de Navarra, Pamplona, Spain; 16 Scientific Library, Fondazione IRCCS Istituto Neurologico Carlo Besta, Milan, Italy; 17 Department of Intensive Care, University Hospitals Leuven and Laboratory of Intensive Care Medicine, Katholieke Universiteit Leuven, Leuven, Belgium; 18 Department of Anesthesiology IRS, Nykøbing F. Hospital, Nykøbing Falster, Denmark; 19 Department of Pediatric Anesthesia, Meyer University Hospital of Florence, University of Florence, Florence, Italy; 20 Department of Anesthesia and Intensive Care, Policlinico San Martino and University of Genoa, Genoa, Italy; 21 Department of Neurosurgery and Neurocritical Care, Los Angeles Medical Center, Ronald Reagan University of California, Los Angeles, CA USA

**Keywords:** EEG, Monitoring, Sedation, Critically ill, Consensus

## Abstract

**Background:**

The use of processed electroencephalography (pEEG) for depth of sedation (DOS) monitoring is increasing in anesthesia; however, how to use of this type of monitoring for critical care adult patients within the intensive care unit (ICU) remains unclear.

**Methods:**

A multidisciplinary panel of international experts consisting of 21 clinicians involved in monitoring DOS in ICU patients was carefully selected on the basis of their expertise in neurocritical care and neuroanesthesiology. Panelists were assigned four domains (techniques for electroencephalography [EEG] monitoring, patient selection, use of the EEG monitors, competency, and training the principles of pEEG monitoring) from which a list of questions and statements was created to be addressed. A Delphi method based on iterative approach was used to produce the final statements. Statements were classified as highly appropriate or highly inappropriate (median rating ≥ 8), appropriate (median rating ≥ 7 but < 8), or uncertain (median rating < 7) and with a strong disagreement index (DI) (DI < 0.5) or weak DI (DI ≥ 0.5 but < 1) consensus.

**Results:**

According to the statements evaluated by the panel, frontal pEEG (which includes a continuous colored density spectrogram) has been considered adequate to monitor the level of sedation (strong consensus), and it is recommended by the panel that all sedated patients (paralyzed or nonparalyzed) unfit for clinical evaluation would benefit from DOS monitoring (strong consensus) after a specific training program has been performed by the ICU staff. To cover the gap between knowledge/rational and routine application, some barriers must be broken, including lack of knowledge, validation for prolonged sedation, standardization between monitors based on different EEG analysis algorithms, and economic issues.

**Conclusions:**

Evidence on using DOS monitors in ICU is still scarce, and further research is required to better define the benefits of using pEEG. This consensus highlights that some critically ill patients may benefit from this type of neuromonitoring.

**Supplementary Information:**

The online version contains supplementary material available at 10.1007/s12028-022-01565-5.

## Introduction

Liberal sedation protocols and clinical scales are commonly used in the intensive care unit (ICU) for level of consciousness evaluation and management of sedative agents in critically ill patients [1–4]. Moderate to deep sedation (Richmond Agitation Sedation Score [RASS] ≤  − 3) may sometimes be necessary to avoid discomfort, improve mechanical ventilation tolerance, provide neuroprotection, and avoid awareness, especially when neuromuscular blocking agents are administered [5–7]. Once consciousness is lost, evaluation of depth of sedation (DOS) through clinical scales is no longer possible, exposing the patient to an increased risk of excessive sedation, which has been associated with several complications, such as delirium, prolonged mechanical ventilation, hemodynamic instability, increased length of ICU and hospital stay, hospital mortality, and long-term cognitive sequelae among survivors [8, 9]. Over the past 2 decades, several so-called processed electroencephalogram (pEEG) monitoring systems have been introduced into clinical practice to monitor the effects of anesthesia, particularly in the operating room. Such monitoring systems predominantly automatically process EEGs recorded from frontal montages (using 2–4 channels) [10]. However, although these new monitors are becoming more widely accepted during general anesthesia, their use in critically ill patients for sedation and global cortical activity monitoring is less common. Even when patients are receiving neuromuscular blocking agents [7], their level of consciousness is reduced or they can develop seizures [11, 12].

Therefore, a multidisciplinary panel of experts was convened to develop consensus-based recommendations useful for the general intensivist on the use of these tools. The panel was requested to provide recommendations on some basic definitions and on four main topics related to pEEG monitoring for patients admitted to the ICU and requiring sedation:

1. Techniques for electroencephalogram (EEG) monitoring: The panel was requested to provide indications on the technical aspects of pEEG monitors, specifically (a) the number of channels required, (b) visualization of a processed or nonprocessed raw EEG trace, (c) types of parameters most frequently evaluated (burst suppression, burst suppression ratio, digital subtraction array, adimensional index of sedation/anesthesia [e.g., bispectral index (BIS), patient state index (PSI), state entropy (SE), quantium consciousness index (qCON)], electromyography [EMG] signals), and (d) artifact occurrence [13, 14].
2. Patient selection: Which patients to be monitored with pEEG in the ICU.
3. Use of pEEG monitors: How to use pEEG monitoring devices in the ICU, artifact identification, interaction between different drugs, and the raw EEG trace and pEEG algorithms.
4. Competency to use, and training the principles of, pEEG monitoring: Discussing the required skills in the analysis of pEEG data and the number and types of monitored cases required to obtain minimum competency in analyzing this type of neuromonitoring.

## Methods

An international multidisciplinary panel of experts, including 21 clinicians involved in monitoring DOS in critically ill patients with competencies in adult neurocritical care and neuroanesthesiology, was selected (Additional file 1). Panelists were assigned four domains (techniques for EEG monitoring, patient selection, use of the EEG monitors, competency and training the principles of pEEG monitoring) and created a list of questions and statements to be presented, and voted on, by the experts through a Delphi web-based consensus. The Delphi rounds were used to reduce the heterogeneity of the different panelists and to obtain the highest consensus and appropriateness. After each round, the ratings were collated, summarized, and analyzed, with the anonymized summary and analysis returned to each panel member before the following round.

All details regarding panel selection, literature search strategy, and Delphi methodology are presented in Additional file 1.

This document should be reviewed after a 5-year time period, or whenever new evidence from the literature can support the change of an existing recommendation.

## Results

Of the 25 experts who were initially invited to join the panel, 21 (84%) agreed and participated. Some definitions on how to monitor DOS were agreed on by the panelists (Additional file 2). Of the 59 statements, 47 (80%) were classified as highly appropriate/appropriate with strong consensus, two (3.3%) were highly inappropriate/inappropriate with strong consensus, three (4.9%) had a weak consensus, two had uncertain appropriateness (3.3%), and in five (8.2%), no consensus was reached. The list of all voted statements with level of agreement and level of consensus is presented in Tables 1, 2, 3 and 4.

**Table 1:** Technology: outcome of Delphi process for technology statements

Statement	DI	Median AS	Stopping rule^a^	Conclusion
1. Processed quantitative EEG is adequate to monitor the level of sedation	0.059	8	Rd 2	Highly appropriate/strong consensus
2. Four or five channels are sufficient if the EEG is used to titrate the level of sedation	0.195	8	Rd 2	Highly appropriate/strong consensus
3. Two channels are sufficient if the EEG is used to titrate the level of sedation	1.53	4	Rd 3	No consensus
4. A frontal montage is adequate when using the EEG to assess the level of sedation in critically ill patients	0.132	8	Rd 2	Highly appropriate/strong consensus
5. It is important to have a monitor with the possibility to change the wave speed and the EEG amplitude scale	0.132	9	Rd 2	Highly appropriate/strong consensus
6. Dimensionless numerical scales of alertness, such as the BIS, PSI, entropy, etc., are adequate to monitor the level of sedation	0.883	5.5	Rd 2	Uncertain appropriateness
7. If using dimensionless numerical scales of alertness, such as the BIS, PSI, entropy, etc., the manufacturer’s target interval for light or deep sedation should be used as clinically indicated	0.374	7	Rd 3	Appropriate/strong consensus
8. It is important to have a continuous colored density spectrogram of the EEG	0.0901	8	Rd 2	Highly appropriate/strong consensus
9. It is important to be able to change the parameters of the spectrogram, such as the power scale	0.031	8	Rd 2	Highly appropriate/strong consensus
10. Burst suppression should be avoided in all circumstances	0.652	7	Rd 2	Appropriate/weak consensus
11. Burst suppression should be avoided in all circumstances unless to control high intracranial pressure	0.492	8	Rd 2	Highly appropriate/strong consensus
12. Suppression ratio is a good parameter to evaluate the presence of burst suppression	0	8	Rd 2	Highly appropriate/strong consensus
13. If burst suppression is used, the suppression ratio should be kept at < 5%	0.175	8	Rd 3	Highly appropriate/strong consensus

List of all voted statements with level of agreement and level of consensus

AS, appropriateness score; BIS, Bispectral Index; DI, Disagreement Index; EEG, electroencephalography; PSI, Patient State Index

^a^Round (Rd) of the Delphi process after which a stopping rule was reached

**Table 2:** Patient selection: outcome of Delphi process for patient selection statements

Statement	DI	Median AS	Stopping rule^a^	Conclusion
1. All patients receiving sedation in the ICU should be monitored with depth of sedation monitors if clinical evaluation is not possible	0.132	9	Rd 2	Highly appropriate/strong consensus
2. There should be separate criteria for elderly patients when considering the use of depth of sedation monitoring	0.372	7	Rd 3	Appropriate/strong consensus
3. Any patient for whom deep sedation (RASS ≤ − 3) is required will benefit from depth of sedation monitoring	0.132	8	Rd 2	Highly appropriate/strong consensus
4. Patients receiving sedation in the ICU should be monitored with depth of sedation monitors only if neuromuscular blockade is used for mechanical ventilation	0.292	3	Rd 2	Inappropriate/strong consensus
5. Depth of sedation monitors should be used continuously, starting as soon as possible after ICU admission	0.009	8	Rd 2	Highly appropriate/strong consensus
6. Patients who receive only light sedation (RASS of 0 or − 2) do not benefit from depth of sedation monitoring	1.67	7	Rd 3	No consensus
7. Depth of sedation monitoring should be used for patients receiving ECMO	0.132	9	Rd 2	Highly appropriate/ strong consensus
8. Patients admitted to the ICU after ROSC following cardiac arrest should receive cEEG monitoring during cooling or if seizures are present	0	9	Rd 2	Highly appropriate/strong consensus

List of all voted statements with level of agreement and level of consensus

AS, Appropriateness Score; cEEG, continuous electroencephalography; DI, Disagreement Index; ECMO, extracorporeal membrane oxygenation; ICU, intensive care unit; RASS, Richmond Agitation Sedation Score; ROSC, recovery of spontaneous circulation

Determined from freehand comments in Rd 1

Although a stopping rule was not reached because the DI improved between Rds 2 and 3 but did not reach < 0.5, this was accompanied by a fall in median appropriateness score, indicating that consensus that the statement is appropriate was highly unlikely

^a^Round (Rd) of the Delphi process after which a stopping rule was reached

**Table 3:** Assessment of sedation: outcome of Delphi process for use of pEEG monitors statements

Statement	DI	Median AS	Stopping rule^a^	Conclusion
1. The level of sedation should be regularly assessed and documented using a validated sedation scoring system	0.007	9	Rd 2	Highly appropriate/strong consensus
2. The desired level of sedation should be identified for each patient and regularly reassessed	0	9	Rd 2	Highly appropriate/strong consensus
3. Doses of sedative agents should be titrated to produce the desired level of sedation	0	9	Rd 2	Highly appropriate/strong consensus
4. pEEG scores vary between patients at the same subjective level of sedation	0.132	8	Rd 2	Highly appropriate/strong consensus
5. Subjective sedation scoring systems are more reproducible than pEEG during light sedation, in which electrical interference due to muscle activity may artificially elevate pEEG values	0.519	7	Rd 2	Appropriate/weak consensus
6. The RASS and SAS are the most valid and reliable sedation assessment tools for measuring quality and depth of sedation in adult ICU patients	0.164	8	Rd 2	Highly appropriate/strong consensus
7. Measures of brain function (BIS, EEG, PSI, or SE) should be used as the primary method to monitor depth of sedation in noncomatose, nonparalyzed critically ill adult patients	0.639	6	Rd 2	Uncertain appropriateness
8. Measures of brain function (BIS, EEG, PSI, or SE) are adequate substitutes for subjective sedation scoring systems	1.53	6	Rd 3	No consensus
9. Measures of brain function (BIS, EEG, PSI, or SE) should be used as the main form of sedation assessment in adult ICU patients who are receiving neuromuscular blocking agents because subjective sedation assessments are unobtainable in these patients	0.132	9	Rd 2	Highly appropriate/strong consensus

List of all voted statements with level of agreement and level of consensus

AS, Appropriateness Score; BIS, Bispectral Index; DI, Disagreement Index; EEG, electroencephalography; pEEG, processed electroencephalography; PSI, Patient State Index; RASS, Richmond Agitation Sedation Score; SAS, Sedation Agitation Score; SE, state entropy

Determined from freehand comments in Rd 1

Although a stopping rule was not reached, these statements were discontinued after Rd 1 because mutually exclusive alternative statements achieved higher appropriateness scores

^a^Round (Rd) of the Delphi process after which a stopping rule was reached

**Table 4:** Competency to use and training the principles of pEEG monitoring: outcome of Delphi process for training and competencies statements

Statement	DI	Median AS	Stopping rule^a^	Conclusion
1. pEEG monitoring should be considered as a specialized specific monitoring technique	0.388	8	Rd 2	Highly appropriate/strong consensus
2 pEEG monitoring competencies are required only by neurointensivists or neuroanesthesiologists	1.53	5	Rd 2	No consensus
3. pEEG monitoring competencies are required by every category of intensivist	0.149	8	Rd 2	Highly appropriate/strong consensus
4. pEEG monitoring competencies are required by every category of anesthesiologist	0.140	8	Rd 2	Highly appropriate/strong consensus
5. For pEEG monitoring in ICU patients, it would be advantageous to define a universal method of implementation and evaluation irrespective of the patient’s characteristics, the sedation used, and whether pharmacological or neurological aspects were considered	0.009	8	Rd 2	Highly appropriate/strong consensus
6. In addition to EEG pattern recognition and the quantitative multiparameter approach, graphical displays of trends and/or spectrograms are useful for intercurrent event or problem detection	0	9	Rd 2	Highly appropriate/strong consensus
7. In addition to EEG pattern recognition and the quantitative multiparameter approach, graphical displays of trends and/or spectrograms are useful for identification of the patient’s neurophysiological status or trends in the neurophysiological status	0	9	Rd 2	Highly appropriate/strong consensus
8. In addition to EEG pattern recognition and the quantitative multiparameter approach, graphical displays of trends and/or spectrograms are useful for setting and adjusting sedative medication	0	9	Rd 2	Highly appropriate/strong consensus
9. In the short term, there is a need for structured fellowship programs to enable acquisition of pEEG monitoring competencies	0.132	9	Rd 2	Highly appropriate/strong consensus
10. In the longer term, pEEG monitoring competencies should be an integral part of postgraduate training programs in intensive care	0.132	9	Rd 2	Highly appropriate/strong consensus
11. Written and/or oral examinations appropriate to evaluate defined learning objectives are an essential component of the assessment of pEEG monitoring competencies	0.164	8	Rd 2	Highly appropriate/strong consensus
12. The successful supervised management of a predefined number of cases is an essential component of the assessment of pEEG monitoring competencies	0.132	9	Rd 2	Highly appropriate/strong consensus
13. Final evaluation of competencies in the clinical setting should include use of a global rating scale	0.132	8	Rd 2	Highly appropriate/strong consensus
14. Training in pEEG monitoring can be successfully delivered entirely in the clinical setting	0.357	7	Rd 2	Appropriate/strong consensus
15. Clinical training in pEEG monitoring should be complemented with “classroom” teaching of the theoretical (physics, neurophysiological, pharmacological, pathological, etc.) aspects	0.132	9	Rd 2	Highly appropriate/strong consensus
16. Rapid recognition of typical patterns of the raw EEG trace at the patient’s bedside aids timely clinical decision-making	0.132	9	Rd 2	Highly appropriate/strong consensus
17. The required competencies for routine pEEG monitoring in ICU are limited to assessment of the effects of sedative medication (pharmaco-EEG and/or pharmaco-pEEG)	0.164	3	Rd 2	Inappropriate/strong consensus
18. pEEG monitoring training would benefit by using the approaches successfully applied to other specialized monitoring/diagnostic techniques, such as transthoracic and/or transesophageal echocardiography	0	8	Rd 2	Highly appropriate/strong consensus
19. Programs of training for pEEG monitoring would benefit from including neurospecialists (neurologist, epilepsy specialist) on the faculty	0.132	9	Rd 2	Highly appropriate/strong consensus
20. New learning resources will need to be developed specifically to support training for pEEG monitoring in the ICU	0.132	9	Rd 2	Highly appropriate/strong consensus
21. The intensivist certified in pEEG monitoring should demonstrate regular continuing professional development activities relevant to pEEG monitoring	0.132	8	Rd 2	Highly appropriate/strong consensus
22. The intensivist certified in pEEG monitoring requires regular recertification in pEEG monitoring	0.164	8	Rd 2	Highly appropriate/strong consensus
23. Recertification of the intensivist certified in pEEG monitoring should be based on review of cases that demonstrate required competencies	0.195	8	Rd 2	Highly appropriate/strong consensus
24. Recertification of the intensivist certified in pEEG monitoring should be based on a written examination	1.61	7	Rd 2	No consensus
25. Recertification of the intensivist certified in pEEG monitoring should be based on review of cases that demonstrate required competencies and a written examination	0.678	8	Rd 2	Highly appropriate/weak consensus
26. In the absence of a system of internal support, external support for the intensivist certified in pEEG monitoring must include the capability for real-time input from a neuro-ICU specialist, neurologist, or neurophysiologist if necessary	0.031	8	Rd 2	Highly appropriate/strong consensus
27. Frequency domain analysis of the EEG signal is useful when monitoring ICU patients	0.009	8	Rd 2	Highly appropriate/strong consensus
28. Time domain analysis of the EEG signal is useful when monitoring ICU patients	0.126	8	Rd 2	Highly appropriate/strong consensus
29. Power domain analysis of the EEG signal is useful when monitoring ICU patients	0	8	Rd 2	Highly appropriate/strong consensus

List of all voted statements with level of agreement and level of consensus

AS, Appropriateness Score; DI, Disagreement Index; ICU, intensive care unit; EEG, electroencephalography; pEEG, processed electroencephalography

^a^Round (Rd) of the Delphi process after which a stopping rule was reached

## Technology

### Question 1: Is pEEG Adequate to Monitor the Level of Sedation?

- We recommend that pEEG should be adequate to monitor the level of sedation (strong consensus).
- We recommend that four or five channels should be sufficient if the EEG is used to titrate the level of sedation (strong consensus).
- We cannot make a recommendation whether two channels should be sufficient if the EEG is used to titrate the level of sedation (no consensus).
- We recommend that a frontal montage should be adequate when using the EEG to assess the level of sedation in critically ill patients (strong consensus).
- We recommend having a monitor with the possibility to change the wave speed and the EEG amplitude scale (strong consensus).

### Background

pEEG provides a compressed and simplified view of the raw EEG signals, allowing for potential evaluation by nonneurophysiologists who can alert the neurophysiologists when required because of matters of concern [13, 15]. Some technical and physiological limitations of these parameters when applied to the bedside for sedation assessment should be considered: pEEG monitoring to guide sedation of patients in intensive care requires specific knowledge to distinguish specific signatures of each drug used for sedation, including the recognition of the features and changes in pEEG values associated with sedation with ketamine, nitrous oxide (faster EEG oscillations, i.e., the beta rhythm, higher index values at loss of consciousness), and dexmedetomidine (profound slow oscillations, i.e., the delta rhythm, low index values in an arousable patient) [15–18]. Nonetheless, integration of pEEG parameters in a wide monitoring platform in addition to the raw EEG can facilitate accurate control of sedation level and differentiate sleep stages [19–22].

Some of the available pEEG monitors have the property of changing the speed of the wave signal. Sometimes, it is helpful to change the speed to a more familiar one to better recognize usual EEG features. The slower speed (10 mm/s) is useful, for example, because low-frequency activity (such as respiration) is better seen at that speed; displaying 20 s or more per page will enhance slow activity and allow analysis of slow periodic complexes or prolonged events, such as seizures.

### Question 2: Are Dimensionless Numerical Scales of Alertness Adequate to Monitor the Level of Sedation?

- We cannot recommend dimensionless numerical scales of alertness (such as the BIS, PSI, entropy, etc.) be adequate to monitor DOS in critically ill patients (uncertain appropriateness, strong consensus).
- We recommend that if using dimensionless numerical scales of alertness (such as the BIS, PSI, entropy, etc.), the manufacturer’s target interval for light or deep sedation should be used as clinically indicated (strong consensus).

### Background

pEEG monitors were launched primarily as “hypnosis monitors” during surgery [10–22]. The use of the output index ranges suggested by the manufacturer for light and deep sedation might be an effective tool as a first approach to guide and individualize sedative drug dosing schemes integrated in a multimodal monitoring strategy in critically ill patients [23–26].

Some limitations of this technology are represented by the influence of EMG/artifacts, reduced signal quality (due to electrode detachment, sweating, or swelling of forehead), and patient conditions (e.g., brain damage) because they can alter the pEEG number [25, 27–31]. Therefore, the pEEG number should be verified by the concordance with the raw EEG rhythm [32, 33].

### Question 3: Is it Meaningful to have a Continuous Colored Density Spectrogram of the EEG?

- We recommend that it should be important to have a continuous colored density spectrogram of the EEG (strong consensus).
- We recommend that parameters of the spectrogram, such as the power scale, should be modifiable (strong consensus).

### Background

It may be challenging for clinicians to interpret a sedation state from the unprocessed raw EEG in real time. With the spectral analysis continuously displayed, it is easier to interpret and recognize changes in the frequency power and to detect small changes in the frequency bandwidth’s structure. Different sedatives acting on different neuronal circuits by different mechanisms have distinct EEG signatures that produce different spectrogram patterns [18]. Propofol-induced unconsciousness, for example, is associated with slow delta and alpha oscillations [33]. EEG spectral patterns in ICU patients have a standardized nomenclature with high interrater agreement and can be a useful tool for EEG screening [34]. Age and comorbidities decrease the EEG amplitude, and weaker power in the alpha band increases the propensity for burst suppression (BS), which is a phenotype of a vulnerable, frail brain [33]. The possibility to adjust the power scale of the spectrogram increases utility because it can increase the visibility of such weak bandwidths.

### Question 4: Should BS be Avoided in All Circumstances?

- We cannot recommend that BS should be avoided in all circumstances (weak consensus).
- We recommend that BS should be avoided in all circumstances unless to control high intracranial pressure (strong consensus).
- We recommend that the suppression ratio (SR) should be a good parameter to evaluate the presence of BS (strong consensus).
- We recommend that if BS is used, the SR should be kept at < 5% (strong consensus).

### Background

BS has been associated with a higher risk of delirium and mortality in critically ill patients [35, 36]. Current guidelines suggest that BIS monitoring appears best suited for sedative titration during deep sedation or neuromuscular blockade, though observational data suggest potential benefit with lighter sedation as well. Sedation that is monitored with the BIS compared with subjective scales may improve sedative titration when a sedative scale is required [37].

It is important to distinguish unintended BS resulting from overdosing of sedative drugs from therapeutic induced BS, which might be potentially useful in situations of low cerebral blood flow and altered metabolism, such as refractory intracranial hypertension and the treatment of refractory status epilepticus. Despite a lack of clear evidence to support this practice and large variability in the degree of EEG suppression achieved, BS remains incorporated in many pragmatic refractory status epilepticus treatment algorithms [38–42].

## Patient Selection

### Question 1: Which Kind of Patients in the ICU Should be Monitored with DOS Monitors?

- We recommend that all patients receiving sedation in the ICU should be monitored with DOS monitors if clinical evaluation is not possible (strong consensus).
- We recommend that separate criteria should be used for elderly patients when considering the use of DOS monitoring (strong consensus).
- We recommend that any patient for whom deep sedation (RASS ≤  − 3) is required should benefit from DOS monitoring (strong consensus).

### Background

The majority of ICU patients require sedation, and previous guidelines [43, 44] suggest the routine use of clinical scales to monitor sedation. These scales are ordinal scales that can accurately assess consciousness levels during mild to moderate sedation, but they are unreliable when consciousness has been lost. Moreover, scoring involves stimulating the patient, which itself alters the level of sedation and patient comfort. We therefore reached a consensus regarding the use of pEEG when sedation scoring is not possible, such as during deep sedation (RASS of − 4 or − 5) [45] or under neuromuscular blockade.

A correlation between pEEG-based index (e.g., BIS, PSI, SE, qCON) values and the administered dose of intravenous and inhalational anesthetic agents has been demonstrated: the progressive deepening of sedation induces a corresponding progressive reduction in pEEG-based index values [46–49]. Current data on pEEG monitoring in critically ill patients in the ICU are less definitive and more controversial, but their use seems to be beneficial for continuous brain monitoring [50].

### Question 2: Should DOS in the ICU be Monitored Only when Patients are Mechanically Ventilated?

- We recommend that DOS monitoring should be used in patients receiving sedation in the ICU not only when neuromuscular blockade is used for mechanical ventilation (strong consensus).

### Background

In a prospective convenience sample, BIS monitoring well matched with clinical evaluation along a wide range of sedation levels in 63 adult ICU patients under ventilatory support: from very deep sedation (sedation agitation score [SAS] = 1, BIS score = 43) to mild agitation (SAS = 5, BIS score = 100) [51]. Similarly, the BIS well described the DOS in 63 mechanically ventilated ICU patients, with average BIS values that well correlated with the SAS (*r*^2^ = 0.21, *p* < 0.001) [52]. Riker et al. [53] found good correlation between the BIS and the SAS (*r* = 0.60, *p* < 0.001) but noted that electromyographic interference could affect the accuracy of the BIS in cardiac patients receiving neuromuscular blocking agents, a group in whom residual electromyographic activity can cause spurious elevations in the BIS score. The BIS seems to be useful in patients receiving muscle relaxants [31], whereas other DOS devices may be used to quantify the level of propofol/sufentanil sedation in ICU patients, but they have not yet been evaluated in ICU patients receiving muscle relaxants [54]. However, it is important to remember that evaluation of brainstem reflexes and response to pain stimulation remains paramount because monitoring systems should be used to supplement and not replace the clinical examination. Furthermore, the BIS is more reliable than the RASS for maintaining a stable sedation status and intracranial pressure in severe traumatic brain injury [55], and it may be a useful adjunctive tool for the objective assessment of the level of consciousness in patients with brain injury [56].

### Question 3: When Should we Start Monitoring DOS in ICU Patients?

- We recommend that DOS monitors should be used continuously, starting as soon as possible after ICU admission when required (strong consensus).
- We recommend that patients who receive only light sedation (RASS of 0 or − 2) should not benefit from DOS monitoring (strong consensus).

### Question 4: Should DOS be Monitored in Patients Receiving Short-Term Deep Sedation for Procedures Performed in the ICU?

- We recommend that patients receiving short-term deep sedation for procedures performed in the ICU should require DOS monitoring (strong consensus).
- We do not recommend DOS monitoring when patients receive only light sedation (RASS of 0 or − 2) (no consensus).

### Background

The ideal timing for initiating DOS monitoring in ICU patients has yet to be investigated, but given the risks of undersedation and oversedation, the experts consider that DOS monitoring should be started as soon as possible when deeper sedation than an RASS of 0 or − 1 is required [4, 43]. Patients admitted to the ICU can require multiple interventions (e.g., central-line placement, bedside tracheostomy, change of burns dressings, thoracic drainage placement) for which deep sedation, analgesia, and sometimes the administration of neuromuscular blocking agents are required.

### Question 5: Should Patients on Venovenous Extracorporeal Membrane Oxygenation or Extracorporeal Life Support/Venoarterial Extracorporeal Membrane Oxygenation Require DOS Monitoring?

- We recommend that DOS monitoring should be used for patients receiving extracorporeal membrane oxygenation (ECMO) (strong consensus).

### Background

Patients on ECMO have numerous risk factors for delirium, such as hypoxia, reduced cerebral perfusion, and the potential for vascular micro emboli. Hyperactive delirium or agitation can be life threatening for these patients (causing a malfunction of the indwelling vascular lines), so a consequent monitoring and a symptomatic therapy of stress, anxiety, delirium, pain, and insomnia is essential to safely achieve a target RASS of 0 [57]. Lack of sedation monitoring could lead to oversedation and, by inducing BS, might affect the patients’ outcomes, thereby limiting the beneficial effect of extracorporeal oxygenation on cerebral metabolism [58].

### Question 6: Should Patients Admitted to the ICU After Recovery of Spontaneous Circulation Following Cardiac Arrest Receive DOS Monitoring if They Require Sedation for Cooling Purposes?

- We recommend that patients admitted to the ICU after recovery of spontaneous circulation (ROSC) following cardiac arrest should receive continuous EEG (cEEG) monitoring during cooling and rewarming or if seizures are present (strong consensus).

### Background

Sedative and analgesic infusions and neuromuscular blocking agents are commonly used during target temperature management (TTM) for comfort, suppression of shivering, and reduction of metabolic activity, but the optimal regimens are unknown, and dosing strategies vary widely [59]. To counteract these effects, different strategies have been proposed, ranging from a high dose of sedatives and analgesics without neuromuscular blockade to a much lower dose with intermittent or continuous neuromuscular blockade [60]. During TTM, observational studies suggest that sedatives and analgesics accumulate because of impaired metabolism, which can delay wakening, confound neurologic assessment, and potentially result in inappropriate withdrawal of life support [61]. For these reasons, optimization of sedation is thought to be essential in the management of patients with critical illness. Current guidelines also recommend the use of cEEG in these patients for seizure management [62, 63].

In addition, there is evidence that during rewarming, there is an increased incidence of interictal epileptiform discharges 2.6 times as likely as compared with the cooling period [64].

However, cEEG is difficult for nonneurologists to interpret and amplitude-integrated EEG, a type of quantitative EEG usually derived from single- or two-channel cEEG recordings, has been suggested in these patients because it is easy to interpret. Furthermore, after ROSC, it has been reported to be useful for predicting neurological outcomes in adult patients post cardiac arrest [65]. The PSI, SR, and BIS values have been demonstrated to be good predictors for early neuroprognostication in patients post cardiac arrest [66, 67].

## Assessment of Sedation

### Question 1: Are pEEG Values Different Between Patients at the Same Subjective Level of Sedation?

- We recommend that pEEG scores should vary between patients at the same subjective level of sedation (strong consensus).
- We recommend that the level of sedation should be regularly assessed and documented using a validated sedation scoring system (strong consensus).
- We recommend that the desired level of sedation should be identified for each patient and regularly reassessed (strong consensus).
- We recommend that doses of sedative agents should be titrated to produce the desired level of sedation (strong consensus).

### Background

Besides the differences in pharmacodynamics and pharmacogenetics in relation to anesthetic and sedative drugs, other factors may influence the EEG signal: cerebral blood flow, hypothermia, age, brain damage, and others. When interpreting the pEEG, all possible causes of patient variability must be taken into consideration, including different EEG spectra during burst and suppression periods [34, 68–70].

### Question 2: Are Subjective Scoring Systems of Sedation More Reproducible than pEEG During Light Sedation, in Which Electrical Interference Due to Muscle Activity May Artificially Elevate pEEG Values?

- We recommend that the RASS and SAS should be the most valid and reliable sedation assessment tools for measuring quality and DOS in adult ICU patients (strong consensus).
- We cannot recommend that subjective sedation scoring systems should be more reproducible than pEEG during light sedation, in which electrical interference due to muscle activity may artificially elevate pEEG values (weak consensus).

### Background

Although pEEG has the advantage of being easily interpreted by doctors and nurses who are not experts in neurophysiology, it is very vulnerable to artifacts caused by electromyographic signals from shivering or facial movements or from interference from electrical signals from nearby machines (body thermoregulating systems, hemofiltration, and ECMO machines) [71]. Efforts to improve artifact detection and removal and signal-to-noise ratios are underway [40, 42, 67, 72, 73]. Until these systems have been validated, subjective sedation scoring systems should be considered more reproducible than pEEG for patients who are lightly sedated and for whom neurological evaluation is possible, particularly when the risks of artifact exposure are high [74, 75].

### Question 3: Should Measures of DOS-Monitoring pEEG (BIS, EEG, PSI, SE) be Used as the Primary Method to Monitor DOS in Noncomatose, Nonparalyzed Critically Ill Adult Patients?

- We cannot recommend that pEEG-derived indexes (BIS, EEG, PSI, SE) should be used as the primary method to monitor DOS in noncomatose, nonparalyzed critically ill adult patients (uncertain appropriateness).

### Background

Although pEEG has the advantage of being easily interpreted by doctors who are not experts in neurophysiology, it is however more subject to artifacts compared with traditional EEG. These artifacts may be due to either nearby devices causing interference (body thermoregulating systems, hemofiltration, and ECMO machines) or muscle activity, such as shivering. Having said this, recent developments regarding this technology are in progress, and efforts are directed toward a further reduction in noise and artifacts [67, 76, 77]. Until these systems have been validated, subjective sedation scoring systems are to be considered more reproducible than pEEG for patients who are lightly sedated and for whom neurological evaluation is therefore possible, particularly when the risk of artifact exposure is high [78, 79].

### Question 4: Are Measures of Brain Function (pEEG) Adequate Substitutes for Subjective Sedation Scoring Systems?

- We do not recommend pEEG-derived indexes (BIS, EEG, PSI, SE) as adequate substitutes for subjective sedation scoring systems (no consensus).

### Background

pEEG devices cannot, and should not, replace the clinically validated scales but rather be supplemental to them because these latter are globally more informative of the clinical sedation status of ICU patients.

### Question 5: Should Measures of Brain Function (pEEG) be Used as the Main Form of Sedation Assessment in Adult ICU Patients who are Receiving Neuromuscular Blockade, Because Subjective Sedation Assessments are Unobtainable in These Patients?

- We recommend that pEEG-derived indexes (BIS, EEG, PSI, SE) should be used as the main form of sedation assessment in adult ICU patients who are receiving neuromuscular blocking agents because subjective sedation assessments are unobtainable in these patients (strong consensus).

### Background

EEG-based monitoring devices are well suited to facilitate sedative titration during deep sedation and especially when neuromuscular blocking agents have been administered (e.g., in patients with acute respiratory distress syndrome, those requiring prone positioning, and those requiring venoarterial and venovenous ECMO) [53, 54, 59].

## Competency to Use and Training the Principles of pEEG Monitoring

### Question 1: Is pEEG Important Enough to Need an Official Structured Teaching and Training?

- We recommend that pEEG monitoring should be considered as a specialized specific monitoring technique (strong consensus).
- We do not recommend that pEEG monitoring competencies be required only by neurointensivists or neuroanesthesiologists (no consensus).
- We recommend that pEEG monitoring competencies should be required by every category of intensivist (strong consensus).
- We recommend that pEEG monitoring competencies should be required by every category of anesthesiologist (strong consensus).
- We recommend that for pEEG monitoring in ICU patients, it should be advantageous to define a universal method of implementation and evaluation irrespective of the patient’s characteristics, the sedation used, and whether pharmacological or neurological aspects are considered (strong consensus).
- We recommend that in addition to EEG pattern recognition and the quantitative multiparameter approach, graphical displays of trends and/or spectrograms should be useful for intercurrent event or problem detection (strong consensus).
- We recommend that in addition to EEG pattern recognition and the quantitative multiparameter approach, graphical displays of trends and/or spectrograms should be useful for identification of the patient’s neurophysiological status or trends in the neurophysiological status (strong consensus).
- We recommend that in addition to EEG pattern recognition and the quantitative multiparameter approach, graphical displays of trends and/or spectrograms should be useful for setting and adjusting sedative medication (strong consensus).
- We recommend that in the short term, there should be a need for structured fellowship programs to enable acquisition of pEEG monitoring competencies (strong consensus).
- We recommend that in the longer term, pEEG monitoring competencies should be an integral part of postgraduate training programs in intensive care (strong consensus).
- We recommend that written and/or oral examinations appropriate to evaluate defined learning objectives should be an essential component of the assessment of pEEG monitoring competencies (strong consensus).
- We recommend that the successful supervised management of a predefined number of cases should be an essential component of the assessment of pEEG monitoring competencies (strong consensus).
- We recommend that final evaluation of competencies in the clinical setting should include use of a global rating scale (strong consensus).

### Background

The use and recourse to technologies, on which clinicians increasingly rely on, requires adequate and appropriate training [78, 80–82]. The experiences reported about university education on the EEG relate mainly to neurology. This teaching almost exclusively concerns one category of doctors in training: residents. Therefore, it is referred to as overspecialization or the acquisition of additional competence at a necessarily advanced level. In this context, modern educational media for learning assistance [83–87] have already led to the publication of a dictionary and interdisciplinary language concerning the EEG as a common basis for work in intensive care [86]. In this area, experiments have already been reported, concerning, on the one hand, doctors in general (including, but not exclusively, emergency doctors, intensivists, and anesthesiologists) [87], and on the other hand, anesthesiology residents. One of the most interesting aspects of this last investigation is that the investigators have really managed to score the provided training [88]. This quantitative aspect appears to be a key point.

### Question 2: Is it Necessary and Preferable to Dispatch a More Holistic Training with Teaching of the Theoretical, Neurological and/or Pharmacological, and Practical Aspects of the pEEG?

- We recommend that the training in pEEG monitoring should be delivered entirely in the clinical setting (strong consensus).
- We recommend that clinical training in pEEG monitoring should be complemented with “classroom” teaching of the theoretical (physics, neurophysiological, pharmacological, pathological, etc.) aspects (strong consensus).
- We recommend that rapid recognition of typical patterns of the raw EEG trace at the patient’s bedside should aid timely clinical decision-making (strong consensus).
- We do not recommend that the required competencies for routine pEEG monitoring in the ICU be limited to assessment of the effects of sedative medication (strong consensus).

### Background

Initially, clinicians require knowledge and experience to comprehend the principle of pEEG monitoring and to interpret the basic EEG waveforms, spectrogram, and processed indices during general anesthesia in the healthy brain and with minimal interference, for which a 35-min training session has been considered sufficient [89]. This should be further implemented by a more extended period of training and experience to understand the influence of other pathologies, conditions, and/or artifacts on the EEG [90, 91]. Finally, clinicians should be taught to be aware of the limits and advantages of each pEEG monitor used. Frequent use of pEEG in the ICU, combined with multidisciplinary teaching, is warranted to improve the performance of clinicians when using pEEG monitoring to manage patients and estimate their prognosis.

The use of interactive teaching approaches and simulation seems to be very effective [92, 93].

### Question 3: Might the pEEG Monitoring Training be Considered Similarly to the Previous Approaches Regarding Different Special Techniques of Monitoring or Diagnosis, Such as as the Transthoracic and/or Transesophageal Echocardiography and Others?

- We recommend that pEEG monitoring training should benefit by using the approaches successfully applied to other specialized monitoring/diagnostic techniques, such as transthoracic and/or transesophageal echocardiography (strong consensus).

### Background

Bombardieri and colleagues [90] reported the application of simulation for training in pEEG and found a significant improvement of clinicians without prior EEG training in identifying EEG waveforms corresponding to different hypnotic depths and also in recognizing when the hypnotic depth suggested by the EEG was discordant with the pEEG index.

### Question 4: Would the Faculty Include Neurospecialists (Neurologist, Epilepsy Specialist, etc.) Regarding not Only Basic but also Advanced Theoretical Aspects of the EEG? Will New Learning Resources Need to be Developed Specifically to Support Training for pEEG Monitoring in the ICU?

- We recommend that programs of training for pEEG monitoring should benefit from including neurospecialists (neurologist, epilepsy specialist) on the faculty (strong consensus).
- We recommend that new learning resources be developed specifically to support training for pEEG monitoring in the ICU (strong consensus).

### Background

The participation of specialists with a neuroscientific background in the faculty of pEEG teaching and training courses provides effectiveness and quality in two respects: firstly, because of their gained knowledge of the EEG signal itself in all its aspects (basic and theoretical, physiological and pathophysiological, neurological and pharmacological) and secondly, because of their more advanced experience regarding EEG training [94–96].

### Question 5: Should the Intensivist Certified in pEEG Monitoring Demonstrate Regular Continuing Professional Development Activities Relevant to pEEG Monitoring?

- We recommend that the intensivist certified in pEEG monitoring should demonstrate regular continuing professional development activities relevant to pEEG monitoring (strong consensus).
- We recommend that the intensivist certified in pEEG monitoring should require regular recertification in pEEG monitoring (strong consensus).
- We recommend that recertification of the intensivist certified in pEEG monitoring should be based on review of cases that demonstrate required competencies (strong consensus).
- We cannot recommend that recertification of the intensivist certified in pEEG monitoring should be based on a written examination (no consensus).
- We cannot recommend that recertification of the intensivist certified in pEEG monitoring should be based on review of cases that demonstrate required competencies and a written examination (weak consensus).
- We recommend that in the absence of a system of internal support, external support for the intensivist certified in pEEG monitoring must include the capability for real-time input from a neuro-ICU specialist, neurologist, or neurophysiologist if necessary (strong consensus).

### Background

Literature is divided regarding the balance between support and continuing education and regular recertification [35]. It is also not clear which authority should be responsible for certification: the academic, regional, or even national level or professional or scientific society [86–88]. The quality of the practice of using any technology to improve patient care and outcomes must be guaranteed. Telemedicine-based solutions have been used with growing effectiveness in high- as well as low- and middle-income countries. How this is implemented will depend on local circumstances [84].

### Question 6: Which Kind of Teaching Should be Discussed During Formal Training?

- We recommend that frequency domain analysis of the EEG signal should be useful when monitoring ICU patients (strong consensus).
- We recommend that time domain analysis of the EEG signal should be useful when monitoring ICU patients (strong consensus).
- We recommend that power domain analysis of the EEG signal should be useful when monitoring ICU patients (strong consensus).

### Background

Graphical representation (trend vs. spectrogram) represents a third reading level of the EEG that should be implemented and simultaneously visible with the raw trace and other derived parameters, such as the pEEG index SR. Graphical representations convey information about the effect of general anesthesia or sedation, the spectral signature of the drug, and patterns associated with the age of the patient [33, 80, 90, 97–101]. Trends or spectrograms can reveal the occurrence of excessive EEG suppression or an increase in activity level consistent with a nonconvulsive seizure episode, both of which are amenable to rapid therapeutic intervention, and specific patterns may help the clinician to assess the patient’s underlying condition and formulate a prognosis (i.e., delta/theta septic encephalopathy, renal or hepatic failure, BS due to severe brain damage) [100–105].

## Discussion and Conclusions

This consensus provides expert-based statements on how pEEG should be used when it is applied to critically ill patients requiring sedation. It recommends that if clinical evaluation is not possible, all deeply sedated patients in the ICU should benefit from DOS monitors. It suggests that specific training programs should be implemented to train the general intensivist in delivering sedation in critically ill patients in an objective manner.

DOS monitoring of critically ill patients remains a challenging topic because of the contradicting results in the literature [106]. The main barriers to the routine use of these monitors in the ICU are represented by (1) the lack of knowledge, especially outside the neurological ICU; (2) the lack of validation of the use of the monitors for prolonged sedation; (3) the lack of a standardization between monitors based on different EEG analysis algorithms; (4) the financial constraints limiting availability of the monitors; and, finally, (5) the unknown effect of excessive sedation on the long-term outcome of these patients. Future applications should be investigated, such as the possible use of machine learning algorithms [11, 107] and the raw trace to predict the probability of refractory/super-refractory status epilepticus, delirium, ICU length of stay, and patients’ outcomes in general.

Limitations of the current consensus are represented by the lack of a systematic review of the literature that could support, with more evidence, the statements presented and by the selection of the panel, which could have led to bias due to conflict of interests. However, the final document was approved by the whole panel of experts, including those not supporting any specific DOS technology or monitors.

This document provides many points that need future discussion. The time has come to design high-quality studies to test the hypothesis regarding the utility and feasibility of using pEEG monitoring in the ICU to prevent delirium and improve patients’ outcomes. Future investments should be aimed at reaching these objectives.

## Supplementary Information

Below is the link to the electronic supplementary material.

Supplementary file1 (DOCX 24 KB)

Supplementary file2 (DOCX 17 KB)

## Data Availability

All data generated or analyzed during this study are included in this published article and its supplementary information files.

## Abbreviations

BIS: Bispectral Index
BS: Burst suppression
cEEG: Continuous electroencephalography
DOS: Depth of sedation
ECLS: Extracorporeal life support
ECMO: Extracorporeal membrane oxygenation
EMG: Electromyography
ICU: Intensive care unit
pEEG: Processed electroencephalography
PSI: Patient State Index
qCON: Quantium Consciousness Index
RASS: Richmond Agitation Sedation Score
ROSC: Recovery of spontaneous circulation
SE: State entropy
SR: Suppression ratio
TTM: Target temperature management
