# Supplementary material for: Processed Electroencephalogram-Based Monitoring to Guide Sedation in Critically Ill Adult Patients: Recommendations from an International Expert Panel-Based Consensus
Source: Neurocrit Care. 2022 Jul 27;38(2):296–311. doi: 10.1007/s12028-022-01565-5 (PMC10090014; doi:10.1007/s12028-022-01565-5)
Supplement: Supplementary file 1 — Supplementary file1 (DOCX 24 KB) [file 12028_2022_1565_MOESM1_ESM.docx]

**Additional file 1.**

**METHODS**

Given the paucity of evidence in the field of the consensus topic, we did not perform a systematic review of the literature.

**Scientific committee**: the project was conceived by two chairpersons with academical experience in the field of pEEG (ML, FR) who established the project’s aims, timeframe and milestones and a methodologist (PH) who selected the experts, identified the area of investigation, and managed the voting process.

**Expert selection**: 25 clinician experts were invited to participate to the project based on: a- their clinical and/or scientific expertise and involvement in neurocritical care, neuroanesthesiology, neurology and general intensive care practice, b- their willingness to be proactive in the project and c- the aim of obtaining an appropriate multidisciplinary representation. However as not all experts have the same competence in the different domains, the whole panel votes all the statements to avoid any bias.

**Question formulation**: Clinical queries were developed in the form of four Population/Intervention/Comparison/Outcome (PICO) groups, further leading to the identification of four clinical domains related to the use of pEEG in adult critically ill patients, which ultimately generated a preliminary list of statements that were submitted to the panelists. The experts decided not to be focused on specific topics related to neurocritical care.

**Literature search strategy:** The literature search strategy was developed by ML, CC, FR and SM (information specialist) on the following electronic databases: Medline via PubMed; the Cochrane Library; Cumulative Index to Nursing and Allied Health Literature (CINAHL); and EMBASE. We included papers published in the period 1 January 2000 – January 2022 in adult population. Combinations of the following subject headings and keywords were used across all databases: “processed EEG” or “continued EEG” and several synonyms in “Critical” or “Intensive Care”. The full electronic search strategies are included in Appendix 1 below in this section. A total of 1169 records were identified. Articles identified this way went through a two round selection process.

The first title and abstract screening removed duplicates and selected papers accordingly with the studies inclusion criteria defined earlier. Systematic reviews, randomized controlled trials, cohort studies, case control studies and cross-sectional surveys were included. Existing guidelines were identified and considered separately. Narrative reviews, editorials, case series or case reports were excluded. Only English language papers and papers describing adults were included. A total of 89 papers were selected.

As we tried to include all relevant papers, the panelists were able to include additional papers relevant to the Consensus but not identified by the literature search. After this selection, only 68 full text papers were identified.

**Delphi method and decision rule**: The modified iterative Delphi process [1] was conducted using online tools. In each round, panel members rated the statements on a 9-point Likert scale with a rating of “1” indicating a completely inappropriate statement through “9” representing a completely appropriate statement. The median rating was used to classify the appropriateness, while the level of consensus was evaluated using the disagreement index (DI), which describes the measure of the spread of ratings with a mathematical adjustment for asymmetry. The questionnaire was submitted to the experts via a web-based survey platform (Google Forms, Menlo Park, California, USA, docs.google.com/forms). After each round, the ratings were collated, summarized and analysed, with the anonymized summary and analysis returned to each panel member before the following round. From round 2 onwards, statements were included within the final, round unless a stopping criterion was reached (DI < 0.5 or the DI failed to improve by > 15% in successive rounds). At the completion of the Delphi process, statements were classified as highly appropriate/inappropriate (median rating ≥ 8), appropriate (median rating ≥ 7 but < 8) or uncertain (median rating <7) and with strong (DI < 0.5) or weak (DI ≥0.5 but < 1) consensus respectively.

An internal review from the panelist and an external review from a non-voting expert (TB) were used to finalize the consensus document.

**References**

1. Hopkins PM, Cooke PJ, Clarke RC, et al: Consensus clinical scoring for suspected perioperative immediate hypersensitivity reactions. Br J Anaesth. 2019;123:e29-e37.

## Appendix 1. Literature search strategy

| **PubMed** ("processed electroencephalogra*"[Title/Abstract] OR "processed EEG"[Title/Abstract] OR "continuous electroencephalogra*"[Title/Abstract] OR "Continuous EEG"[Title/Abstract] OR "cEEG"[Title/Abstract] OR "pEEG"[Title/Abstract])) AND (("Critical Care"[MeSH Terms] OR "Critically Ill"[Title/Abstract] OR "Critical Illness"[MeSH Terms] OR "Intensive Care Units"[MeSH Terms] OR "ICU"[Title/Abstract]) NOT (“animals"[MeSH Terms] not “humans"[MeSH Terms])  NOT (“child”[MeSH Terms] OR “infant”[MeSH Terms] OR “adolescent”[MeSH Terms]) not “adult” [MeSH Terms]  Filters: Publication date from 2000/01/01 to 2022/01/24  **481 records**  ("processed electroencephalogra*"[Title/Abstract] OR "processed EEG"[Title/Abstract] OR "continuous electroencephalogra*"[Title/Abstract] OR "Continuous EEG"[Title/Abstract] OR "cEEG"[Title/Abstract] OR "pEEG"[Title/Abstract])) AND (("Critical Care"[MeSH Terms] OR "Critically Ill"[Title/Abstract] OR "Critical Illness"[MeSH Terms] OR "Intensive Care Units"[MeSH Terms] OR "ICU"[Title/Abstract]) Filters: from 2000 - 2022  **421 records**  ("Electroencephalography"[MeSH Terms] OR "electroencephalogra*"[Title/Abstract] OR "EEG"[Title/Abstract] OR "pEEG"[Title/Abstract] OR "processed electroencephalogra*"[Title/Abstract] OR "Continuous EEG"[Title/Abstract] OR "continuous electroencephalogra*"[Title/Abstract] OR "cEEG"[Title/Abstract] OR "quantitative EEG"[Title/Abstract] OR "monitoring, physiologic"[MeSH Terms] OR "Neurophysiological Monitoring"[MeSH Terms] OR "Consciousness Monitors"[MeSH Terms] OR "bi-spectral"[Title/Abstract] OR "bispectral index*"[Title/Abstract] OR "Bispectral"[Title/Abstract] OR "consciousness monitor*"[Title/Abstract] OR "spectral entropy"[Title/Abstract] OR "spectral edge"[Title/Abstract] OR "patient state index"[Title/Abstract] OR "PSI"[Title/Abstract] OR "state entropy"[Title/Abstract] OR "qCON"[Title/Abstract]) AND ("Critical Care"[MeSH Terms] OR "Intensive Care"[Title/Abstract] OR "critical illness*"[Title/Abstract] OR "Critically Ill"[Title/Abstract] OR "Critical Illness"[MeSH Terms] OR "Intensive Care Units"[MeSH Terms] OR "ICU"[Title/Abstract] OR "anesthesia recovery period"[MeSH Terms]) AND ("Deep sedation" [Mesh] OR sedation*[Title/Abstract] OR "deep sedation"[Title/Abstract] OR "sedative*"[Title/Abstract]) AND ((alladult[Filter]) AND (2000:2022[pdat]))  Filters: Adult: 19+ years, from 2000 – 2022/01/23 | **Embase**  1 exp processed electroencephalogra*/ or exp processed EEG* or pEEG).mp.  2 exp continuous EEG ).mp.  3 exp critical care (critically ill or critical illness or intensive care unit* or (ICU)).ti,ab.  4 1 and 2 and 3  5 exp animal/ not exp human/  6 4 not 5  7 exp juvenile/ not exp adult/  8 6 not 7 (  9 limit 8 to yr="2010 -Current"  #15. #12 AND ([adult]/lim OR [aged]/lim OR [very **746**  elderly]/lim) AND [2000-2022]/py AND [humans]/lim  #14. #12 AND ([adult]/lim OR [aged]/lim OR [very 761  elderly]/lim) AND [2000-2022]/py  #13. #12 AND ([adult]/lim OR [aged]/lim OR [very 780  elderly]/lim)  #12. #10 AND #11 1,694  #11. #1 OR #2 OR #3 3,361  #10. #4 OR #5 OR #6 OR #7 OR #8 OR #9 1,287,294  #9. 'critical care' OR 'intensive therap*':ti,ab 437,652  #8. 'critically ill*':ti,ab 77,794  #7. 'critically ill patient'/exp 55,126  #6. 'intensive care unit'/exp OR icu:ti,ab 306,273  #5. 'critical illness'/exp OR 'critical ill':ti,ab 33,486  #4. 'intensive care'/exp 801,292  #3. 'processed eeg' OR 'processed 488  electroencephalogra*' OR 'peeg':ti,ab  #2. 'continued eeg' OR 'continuing 2,848 electroencephalography' OR ceeg OR 'continuous  eeg' OR 'continuous electroencephalography':ti,ab  #1. 'continuous electroencephalography'/exp 159 |
| --- | --- |
| Cochrane Database of Sistematic Reviews("continued EEG"):ti,ab,kw OR ("continuous EEG"):ti,ab,kw OR ("continuous electroencephalography"):ti,ab,kw OR ("processed electroencephalography"):ti,ab,kw OR ("processed EEG"):ti,ab,kw" **2 RS 164 Trials** | **Cochrane Central Register of Controlled Trials**  (“continued EEG"):ti,ab,kw OR ("continuous EEG"):ti,ab,kw OR ("continuous electroencephalography"):ti,ab,kw OR ("processed electroencephalography"):ti,ab,kw OR ("processed EEG"):ti,ab,kw AND ("Critical Care" OR "Intensive Care" OR "critical illness" OR "Critically Ill" OR "Intensive Care Units" OR "ICU"):ti,ab,kw  **52 Trials 0 RS** |
