# Supplementary material for: Processed Electroencephalogram-Based Monitoring to Guide Sedation in Critically Ill Adult Patients: Recommendations from an International Expert Panel-Based Consensus
Source: Neurocrit Care. 2022 Jul 27;38(2):296–311. doi: 10.1007/s12028-022-01565-5 (PMC10090014; doi:10.1007/s12028-022-01565-5)
Supplement: Supplementary file 2 — Supplementary file2 (DOCX 17 KB) [file 12028_2022_1565_MOESM2_ESM.docx]

**Additional file 2.** Definitions of most repeated terminology.

| Term | Definition |
| --- | --- |
| Depth of sedation (DOS) | monitoring by means of processed EEG-based algorithms consists of a combination of information that includes: raw EEG trace, numeric scales which represent the different levels of patient unconsciousness, density spectral array representation, additional data (burst-suppression rate, spectral edge frequency, artefacts, electromyographic signal). |
| Electroencephalography (EEG) | Method or technique used to record electrical signals of the brain. The signal that is recorded is the electroencephalogram. |
| Quantitative EEG (QEEG) | is the mathematical processing of digitally recorded EEG in order to highlight specific waveform components, transform the EEG into a format or domain that elucidates relevant information, or associate numerical results with the EEG data for subsequent review or comparison.  Signal analysis is the quantitative measurement of specific EEG properties or a transformation of the raw, digitally recorded EEG signal into numerical parameters other than the traditional amplitude versus time [1,2].  EEG can be quantified in terms of its amplitude, power, frequency and rhythmicity in order to generate numerical values, ratios or percentages; graphically display arrays or trends; and set thresholds for alarms.  Several quantitative EEG measures have been used: calculation of power within different frequency bands; ratios or percentages of power in specific frequency bands; and spectral edge frequencies (based on the frequency under x % of the EEG resides). |
| Processed EEG (pEEG) monitoring | is the result of a mathematical and statistical modelling applied to the digitalized raw EEG data to derive a depth-of-anesthesia / depth-of-sedation measurement. The analysis involves EEG feature extraction and inclusion into a final dimensionless index correlated with a range of arousal levels.  To extract the relevant EEG features, the different algorithms use time domain (examining the EEG signal as a function of time, for example zero-order frequency or wavelet analysis) and frequency domain analysis methods (analyzing signals as a function of frequency using Fourier Transformation and Bispectral Analysis) [2-4]. |
| Continuous EEG monitoring (cEEG) | This involves monitoring using a standard 21 electrode or simplified (usually 8 or 10 electrode) montage to record and display multiple channels of EEG. This technique allows uninterrupted assessment of cerebral cortical activity with good spatial and excellent temporal resolution. This technique provides a means of constantly assessing brain function in critically ill obtunded and comatose patients. |
| SR (Suppression Ratio). | It is a main additional parameter indicating the fraction of time, in a given time interval, that the EEG is suppressed or isoelectric, meeting the criteria for suppression (e.g. Voltage < 5 µV, > 0.5 sec [5]. It’s usually expressed as a number between 0 (absence of suppression) and 100 (EEG fully suppressed). Its value should be always 0 since higher values indicate excessive over sedation or severe brain damage. The number is usually increased during Burst Suppressions where the EEG shows periods of isoelectricity alternated to a burst of high voltage activity. |
| DSA (Density Spectral Array). | DSA is a color graphic display that represents the EEG frequencies (as identified on the y-axis) and their power expressed by a color ranging from blue (minimum power) to dark red (maximum power) calculated throughout the time (placed on x-axis). The DSA is derived from the analysis of the EEG in the frequency domain, by mean of the Fast Fourier analysis (FT), a highly complex mathematical method. FT deconstructs the original raw EEG signal into the sinusoidal waves of different frequencies (cycles per second, Hz) i.e. the gamma (> 20 Hz), beta (12-20 Hz), alfa (8-12 Hz), theta (4-8 Hz) and slow/delta (0.5-4 Hz) wave composing the EEG. Moreover, for each wave, it calculates the amplitudes and relative power (expressed in dB) associated (defined as power spectrum) [4]. With the increase in the hypnotic effect, the EEG frequencies shift from rapid (gamma and beta) to slower rhythms (delta+alfa like, and theta) and, consequently, the whole power of the EEG is expressed mainly by the lower frequencies. Therefore, at Loss of consciousness (LOC), the DSA display a dark red color (maximum power) mainly at the lower frequencies of the spectrum (below 12 Hz). [6] |
| MEF (Median Edge Frequency) and SEF (Spectral Edge Frequency) | are additional values calculated in frequency domain and their unit are the Hz. MEF is the frequency of a power spectrum at which 50% of the power is at lower frequencies and 50% of the power is at higher frequencies. SEF is the frequency below which 95% of the total spectral power is located [6,7]. They are both usually displayed on the DSA as continuous white lines during the time. They are clinically useful for tracking whether spectrogram power is shifting to lower or higher frequencies. At LOC, SEF is usually < 12 Hz and MEF < 4-5 Hz [7] |

**References**

1. Nuwer M Assessment of digital EEG, quantitative EEG and EEG brain mapping. Neurology 1997; 49:277-292.

2. Isley M, Edmonds HL, Stecker M. Guidelines for intraoperative neuromonitoring using raw (analog or digital waveforms) and quantitative electroencephalography: a position statement by the american society of neurophysiological monitoring. J Clinical Monit Comput 2009; 23:369-390.

3. Teplan M. Fundamentals of EEG measurement. Measurement Sci Rev 2002; 2:1-11.

4. Rampil IJ. A primer for EEG signal processing in anesthesia. Anesthesiology. 1998; 89:980-1002.

5. Bombardieri AM, Wildes TS, Stevens T, et al. Practical Training of Anesthesia Clinicians in Electroencephalogram-Based Determination of Hypnotic Depth of General Anesthesia. Anesth Analg 2020;130:777-786.

6. Fahy BG, Chau DF. The technology of processed electroencephalogram monitoring devices for assessment of depth of anesthesia. Anesth Analg. 2018;126:111–7.

7. Purdon PL, Sampson A, Pavone KJ, et al. Clinical Electroencephalography for Anesthesiologists: Part I: Background and Basic Signatures. Anesthesiology. 2015;123:937-960.
